# Nanoparticles in the Environment and Nanotoxicology

**DOI:** 10.3390/nano13061053

**Published:** 2023-03-15

**Authors:** Vivian Hsiu-Chuan Liao

**Affiliations:** Department of Bioenvironmental Systems Engineering, National Taiwan University, Taipei 106, Taiwan; vivianliao@ntu.edu.tw; Tel.: +886-2-33665239

Nanomaterials, including engineered nanoparticles and microplastics/nanoplastics, have attracted increasing concern as they might potentially release into the environment, leading to potential risks to ecosystems. However, there are still huge gaps and unknown knowledge about the fate, behavior, and toxicity of nanoparticles in the environment. This Special Issue aims to provide recent novel research findings of nanoparticles on their determination, detection, and degradation in the environment as well as the toxicity of nanoparticles. This Special Issue provides ten outstanding papers comprising two review articles and eight research articles with a range of original contributions detailing various types of nanoparticles in terms of their toxicity [1,2,3,4,5], removal of antibiotics [2,6], and behavior in natural environments [3,7,8].

This Special Issue collected various research papers addressing the toxic effects of various types of nanoparticles using different tested organisms in various media. For example, How and Huang [1] utilized the soil nematode *Caenorhabditis elegans* to investigate neurotoxicity caused by zinc oxide nanoparticles (ZnO-NPs) exposure. They reported that ZnO-NPs particulates attribute to the neurotoxicity in *C. elegan*s via dietary transfer [1]. Le et al. [4] synthesized nano-sized artificial black carbon (aBC), examining its toxicity on A549 human lung cells, and found that aBC with an increased content of the oxygen functional group displayed higher toxicity to A549 cells. Interestingly, exposure to Ce oxide nanoparticles (nCeO_2_), even at high concentrations, did not result in negative effects on spontaneous plant species, the monocot *Holcus lanatus* and dicots *Lychnis-flos-cuculi* and *Diplotaxis tenuifolia* [5]. However, studies on the effects resulting from the exposure of terrestrial species to nanomaterials are limited, and this study highlights the importance of research in this field.

It is worth noting that the behavior of engineered nanoparticles in natural aquatic environments plays a vital role in determining their toxicity and associated risks [9]. Lee et al. [3] investigated the behavior of silver nanoparticles (Ag-NPs) and ZnO-NPs in natural waters to determine their toxicity in zebrafish embryos. They observed that the acute toxicity of AgNPs or ZnONPs in nature water was much lower than that of Milli-Q (MQ) water [3]. This suggests a possible interaction and transformation between AgNPs or ZnONPs and components in the natural water environment, leading to reduced toxicity [3]. The continuous flow dissolution method was established to measure dissolution rates of Ag-NPs in environmentally relevant water [7]. This work might support the Organization for Economic Co-operation and Development (OECD) testing guidelines focusing on natural aquatic environments [7]. Furthermore, a wide range of commercial products containing nano-enabled products (NEPs) were assessed for the release of engineered nanomaterials (ENMs) and their characteristics in environmental waters [8]. 

The contamination of pharmaceuticals and personal care products (PPCPs) has driven attention due to their intensive usage and widespread into the environment as well as their potential risk to humans and ecosystems [10]. In this Special Issue, the potential of using nanomaterials to remove PPCPs was reported [2,6]. Capsoni et al. [2] synthesized halloysite nanotubes (HNT) with magnetic Fe_3_O_4_ nanoparticles as adsorbents to quantitatively reduce the concentration of antibiotic ofloxacin in tap, river, and effluent waters and further evaluated the acute toxicity with the freshwater organism *Daphnia magna*. Sulfamethazine (SMZ), one of the most commonly used antibiotics, was found to experience degradation that could be promoted by graphene oxide (GO) under UV light in a water environment [6].

Besides engineered nanoparticles, ubiquitous microplastics/nanoplastics have gained concern regarding their distribution, source, and fate in the environment, as well as the toxicity of these plastics to the environment and humans. Yang et al. [11] summarized the distribution, source, occurrence, and fate of microplastics in the atmosphere, which was less reviewed compared to that in oceans, freshwater, and soil. The possible health impacts of microplastics/nanoplastics on humans were viewed by Yee et al. [12]. Currently, the distribution and concentrations of microplastics/nanoplastics in the environments are largely unknown due to the restriction of the detection limit, the lack of validated methods, and no effective methods for the determination and quantification [12,13,14]. This makes an accurate assessment of human health and ecological risks of microplastics/nanoplastics a scientific challenge. Nevertheless, in-depth future research should continue to seek to understand the mechanisms of toxicity and potential risks of chronic exposure to various types of nanoparticles at relevant concentrations in the environment.
